# Transcranial Electrical Stimulation to Enhance Cognitive Performance of Healthy Minors: A Complex Governance Challenge

**DOI:** 10.3389/fnhum.2017.00142

**Published:** 2017-03-27

**Authors:** Jantien W. Schuijer, Irja M. de Jong, Frank Kupper, Nienke M. van Atteveldt

**Affiliations:** ^1^Athena Institute, Faculty of Earth and Life Sciences, VU UniversityAmsterdam, Netherlands; ^2^Department of Educational Neuroscience, Faculty of Behavioral and Movement Sciences, VU UniversityAmsterdam, Netherlands; ^3^Institute of Brain and Behavior Amsterdam, VU UniversityAmsterdam, Netherlands

**Keywords:** Transcranial Electrical Stimulation, cognitive enhancement, child wellbeing, complex problems, governance, responsible innovation, ethics

## Abstract

An increasing number of healthy adolescents are consuming products that can enhance their cognitive performance in educational settings. Currently, the use of pharmaceuticals is the most widely discussed enhancement method in the literature, but new evidence suggests that other methods based on Transcranial Electrical Stimulation (tES) also have potential as cognitive enhancer. Just like pharmaceutical enhancers, the availability and education-related use of tES-devices raise a broad range of ethical, legal, and societal issues that need to be addressed by policy-makers. Few studies, however, have specifically explored these issues in relation to child wellbeing. In this narrative review with systematic search, we describe the issues for child wellbeing that could arise from the availability and education-related use of tES-based enhancers by healthy minors. We demonstrate that the issues form a complex web of uncertainties and concerns, which are mainly incited by two factors. First is the high level of factual uncertainty due to gaps in empirical evidence about the exact working mechanisms and efficacy of tES. Moreover, a lack of insight into the technique’s (long-term) effects on healthy developing brains, and uncertainties about potential cognitive trade-offs have fueled concerns about the technique’s safety and impact. The second factor that contributes to the complexity of issues is the presence of moral diversity in our society. Different opinions exist on whether a certain enhancement effect would be desirable and whether potential risks would be acceptable. These opinions depend on one’s moral perspective, and the way one interprets and weights values such as the child’s autonomy and authenticity. The challenge for proper governance resides in the design of an appropriate framework that is capable of balancing the different moral perspectives in society, while recognizing the uncertainties that still exist. We therefore argue for a responsible innovation approach, which encourages an adaptive attitude toward emerging knowledge and dynamic societal values, to deal with the identified issues regarding tES-based enhancement appropriately.

## Introduction

In educational environments replete of academic performance pressures and competition to secure future career opportunities, students constantly search for ways to improve themselves and to stand out of the crowd. It may therefore not be surprising that the topic of cognitive enhancement has become increasingly popular over the past few years. One of the most widely discussed cognitive enhancers is the so-called “smart pill." Although developed for treating disorders, previous studies have reported that a considerable number of healthy students make use of pharmaceutical products, such as the ADHD drugs methylphenidate (Ritalin, Concerta) and amphetamine salts (Adderall) to enhance their cognitive abilities and improve their educational performances. Reported prevalence rates of these practices vary between studies, which could be related to the differences in study samples, employed definitions, and reporting styles (e.g., life-time prevalence versus past year prevalence). Yet, the numbers do suggest that the consumption of enhancing pharmaceuticals is not uncommon in educational settings. Reported rates in the United States and Canada range from 2.5% to as high as 55% (Smith and Farah, 2011). Although a recent review of Maier and Schaub (2015) suggested that the non-medical consumption of drugs to enhance cognitive performance seems less common in Europe than in the United States, its reported use is still substantial, with disclosed prevalence rates between 2 and 16%. What is particularly striking is that enhancing pharmaceuticals are not only illicitly consumed by adult students in universities, but that some healthy minors in school settings also seem to make use of these substances. For example, a recent study in Switzerland showed that 9.2% of the 16- to 19-year-old secondary school students included in the sample (*n* = 1139) had illicitly used prescription drugs for cognitive enhancement purposes at least once (Liakoni et al., 2015). This suggests that we should not only pay attention to cognitive enhancement practices in college and university settings, but also focus on enhancement behaviors of children in school.

Although most of the enhancers currently used in educational settings are pharmaceuticals, new cognitive enhancement methods based on non-invasive neurotechnologies have emerged that may also find their way into schools. One of these methods is Transcranial Electrical Stimulation (tES). Several recent studies have shown that tES-based technologies, such as transcranial direct current stimulation (tDCS) and transcranial random noise stimulation (tRNS), are not only effective for the improvement of disorder related impairments, but can also be used to induce cognitive enhancement in healthy people (Cohen Kadosh et al., 2010; Cappelletti et al., 2013; Martin et al., 2014; Zwissler et al., 2014). The use of tES techniques has been linked to improvements in several cognitive domains, including memory, attention, language, mathematics and decision-making (Cohen Kadosh, 2013). In some cases, enhancement effects have shown to be long-lasting. A study performed by Cohen Kadosh et al. (2010), in which tDCS was applied to the parietal lobes of healthy adults during training sessions with artificial numerical characters, showed that stimulation of these brain areas resulted in long-lasting enhancement of numerical proficiency. Similarly, a more recent tRNS study demonstrated that concurrent stimulation of the parietal lobes during training of a numerosity discrimination task could boost long-term task performance, with discernable effects up to 16 weeks (Cappelletti et al., 2013). In contrast to Cohen Kadosh et al. (2010), who found the enhancement effects to be specific to learned material only, Cappelletti et al. (2013) demonstrated that the enhancement effects were also transferable to performance on other tasks that measured similar underlying constructs. This would make the technique even more attractive as a tool for enhancement of cognitive functions.

The promising results of enhancement studies have made tES-based neurotechnologies potentially interesting for use in educational settings. However, some studies suggest that positive stimulation effects of tES are not always guaranteed. Both Cohen Kadosh et al. (2010) and Zwissler et al. (2014), for example, showed that reversing the current stream of tES yielded opposite effects on cognitive performance (i.e., an impairment instead of improvement). Moreover, the outcomes of two extensive systematic reviews by Horvath et al. (2015a,b) demonstrated that we do not yet fully comprehend the working mechanisms of tES-based enhancement, as no reliable evidence was found for either cognitive or physiologic effects from tDCS. Further doubts about the effects and working mechanism of tES were fueled by a recent experiment of György Buzsáki and Antal Berényi^1^. These two researchers applied tDCS to the skull of a human cadaver, and found that almost none of the current actually entered the brain. However, the results of that particular experiment have not been peer-reviewed yet, and questions may be raised about the generalizability of results from cadaver studies to living human beings. Recently, several animal studies have been performed to gain more insight into how tES modulates neural function (Bennabi et al., 2014). Translational studies may help to shed light on the exact working mechanisms of tES in the future.

Besides the discussion about the technology’s potential and effectiveness, it is crucial to consider the ethical, legal, and societal issues associated with the application of tES. Various authors have expressed their concerns on these points (Bostrom and Sandberg, 2009; Cohen Kadosh et al., 2012; Maslen et al., 2014b), and this has triggered discussion on the desirability of tES-based enhancing technologies. The pro- and counter arguments provided in this discussion are part of a more extensive and overarching debate on the use of neuroenhancers in general (“the neuroenhancement debate”), including pharmaceutical neuroenhancers. Although many arguments have been put forward that either encourage or criticize the use and availability of neuroenhancers, consensus on the topic has not been reached yet, especially not in regards to the newer enhancing neurotechnologies, such as tES. Moreover, despite the indications that neuroenhancement might already be used before adolescents enter higher education, few studies have specifically explored the issues generated by tES in relation to the child.

This gap in the neuroenhancement literature calls for an analysis of the arguments in the neuroenhancement debate that are applicable to neurotechnologies. Particularly, in light of incorporating a broad perspective on ethical, legal, and societal issues, it would be appropriate to focus on the child’s wellbeing, which is a concept that moves beyond measures that purely relate to brain functioning and cognitive performance levels. Therefore, a first aim of this article is to describe the issues for child wellbeing that could arise from the availability and education-related use of tES-based enhancers by healthy minors. We will do this by using a child wellbeing framework to combine insights from (1) ethical literature on tES-based enhancement, and (2) ethical literature on pharmaceutical enhancement that specifically adopts a child-centered perspective using a narrative review approach with a systematic search (see Supplementary Materials for search strategy).

Identifying child wellbeing issues is essential for our second goal of exploring how to deal responsibly with the availability and education-related use of tES technologies. This question actually relates to a governance challenge, and is particularly relevant to address considering the fact that tES-devices are currently still unregulated, and are therefore relatively easy to access (Maslen et al., 2014a). One only has to internet search “tDCS device” to find out that existing uncertainties about the effects of tES have not tempered the public’s curiosity for tES-based enhancement. A large number of websites and online forums exist that discuss how to build and apply tES-devices at home, with some of these websites having over several thousands of subscribers. In addition, various tES-devices have been launched on the consumer market (e.g., Thync, Foc.us, The Brain Stimulator, ApeX), all of which claim to improve attention, performance or other cognitive functions. Since the devices are portable and relatively inexpensive, with prices ranging from approximately $49 to $299 (Wexler, 2015), they might be particularly alluring to children and parents who would like to boost educational performances. We believe that it is important to specifically address the governance challenge of tES in light of the issues for child wellbeing, since the child-perspective adds a layer of complexity that governance measures should be able to account for. Ethical issues can be regarded as extra sensitive and morally problematic when linked to the stake of children, and this stresses the need for a governance approach that recognizes and deals responsibly with the high complexity of issues associated with tES-based enhancement by minors.

## Enhancement and the Child Wellbeing Perspective

Before we start describing the issues for child wellbeing that arise from the availability and education-related use of tES-devices, we need to clarify the concept of child wellbeing and point out its relevance for discussing the topic of cognitive enhancement. We will first elaborate on this latter term, since the word “enhancement” seems to be used in different ways by different authors that contribute to the scientific discussion on the use of neuroenhancers. In experimental studies, the term ‘enhancement’ is often used as an equivalent of improved performance on specific neuropsychological tests (Schleim, 2014). In contrast, some researchers who study the ethical aspects of neuroenhancement criticize the use of this definition in the neuroenhancement debate, as it may evoke the false assumption that improved test-performance or increased cognitive functionality automatically leads to a better life (Earp et al., 2014; Nagel, 2014; Schleim, 2014). Instead, they opt for a broader and more general definition of enhancement proposed by Savulescu et al. (2011) – also referred to as the welfarist definition – which describes enhancement as: “*any change in the biology or psychology of a person which increases the chances of leading a good life in a given set of circumstances”* (p. 6). An important aspect of this welfarist definition is that it defines enhancement in the context of wellbeing (Savulescu et al., 2011). So, it is not an increase in cognitive functioning that determines whether or not one can speak of enhancement, but whether a change in functioning – which could be either a diminishment or an increase – actually results in heightened levels of overall wellbeing (Earp et al., 2014).

The welfarist approach of enhancement provides a useful starting point for this review, in particular because we focus on the availability and education-related use of neuroenhancing technologies in *healthy* children. Healthy children do not suffer from mental or bodily impairments that they wish to improve in order to reach normal levels of functioning. Instead, healthy children would employ neuroenhancers to perform “better than well” (Elliot, 2003 in Nagel, 2014). However, is performance that is better than well also favorable for each individual child? According to several authors, it requires a holistic approach to answer this question; we need to move beyond the scope of mental and bodily functioning by including aspects that are related to an individual’s surrounding context (Nagel, 2014; Schleim, 2014). The wellbeing perspective of the current study allows us to apply such a holistic approach and to study the effect of the availability and use of neuroenhancing technologies within various life domains of the child.

In literature on the concept of wellbeing, it is widely recognized that children should be treated as a distinct group with their own set of needs and wishes to establish wellbeing (Fattore et al., 2006; Ben-Arieh, 2000). This seems fair, as children differ from adults in several ways, including the level of dependence on family or caregivers and the stage of important mental and physical development. Although many articles have been published that specifically target *child* wellbeing, no consensus has been reached yet on the precise definition of the concept. The Organization for Economic Co-operation and Development (OECD) refers to child wellbeing as a measure for “*the quality of children’s lives*” (Organization for Economic Co-operation and Development [OECD], 2009, p. 24), but acknowledges the lack of a comprehensive framework for the assessment of such quality. Current studies often recognize that the concept child wellbeing consists of multiple dimensions such as mental, social, and physical ones, but a unified view on the number and types of dimensions that should be included has not emerged as yet (Pollard and Lee, 2003; Organization for Economic Co-operation and Development [OECD], 2009; Ben-Arieh and Frones, 2011; Lee, 2014).

**FIGURE 1:**
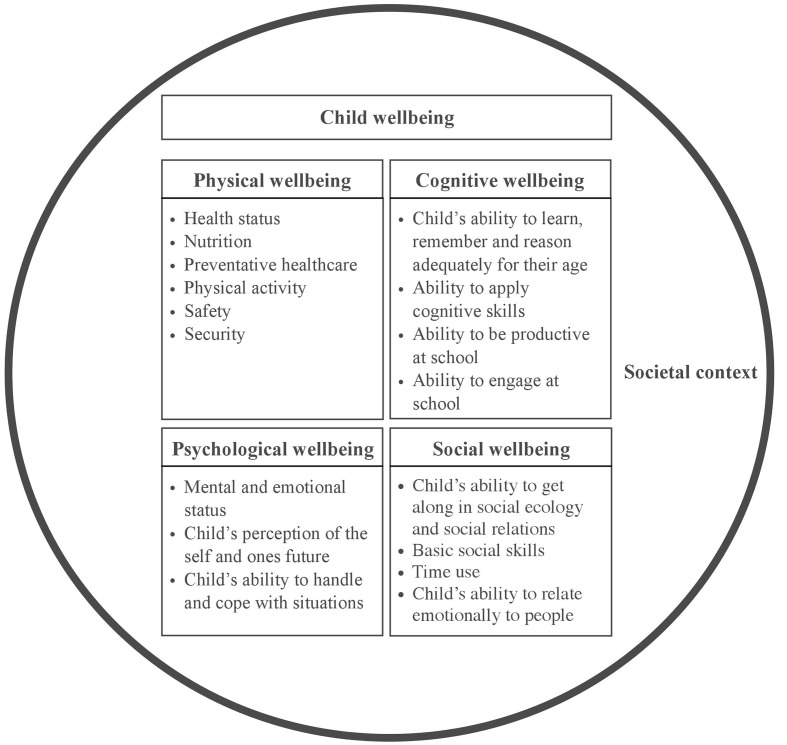
**A framework of child wellbeing, adapted from Moore and Theokas (2008) and Lee (2014)**. The components of each of the child wellbeing domains are explained in the four squares, and the circle around the squares represents the societal context that directly or indirectly influences the domains.

Despite the lack of a universally acclaimed definition and approach to measure child wellbeing, some salient and overarching dimensions of the concept can be recognized when analyzing the various frameworks that have been used in the past. Both Moore and Theokas (2008) and Lee (2014) performed such an analysis and discriminated four main outcome domains of child wellbeing: physical, psychological, cognitive wellbeing, and social wellbeing (see **Figure 1**). In addition, they elaborate on the relevance of studying contextual factors that could indirectly influence the outcome domains of child wellbeing. Moore and Theokas (2008), for example, refer to contexts such as family, peers, school, and neighborhood. Here, we chose to use the term “societal context” to encapsulate all social structures and dynamics in the child’s environment that could impact the level of child wellbeing either positively and negatively. In **Figure 1**, we have depicted the societal context as a circle around the four child wellbeing domains to emphasize its diffuse influence on all these domains. Although both Moore and Theokas (2008) and Lee (2014) stress that their child wellbeing framework is not meant to be all-inclusive, the authors do emphasize its functionality as a basic structure to shape discussion about desired child wellbeing outcomes. In the current article, we use the four child wellbeing outcome domains and the umbrella domain of socio-contextual influences to describe the issues of tES-based cognitive enhancement from a child wellbeing perspective.

## tES-Based Enhancement: Identifying the Issues for Child Wellbeing

**FIGURE 2:**
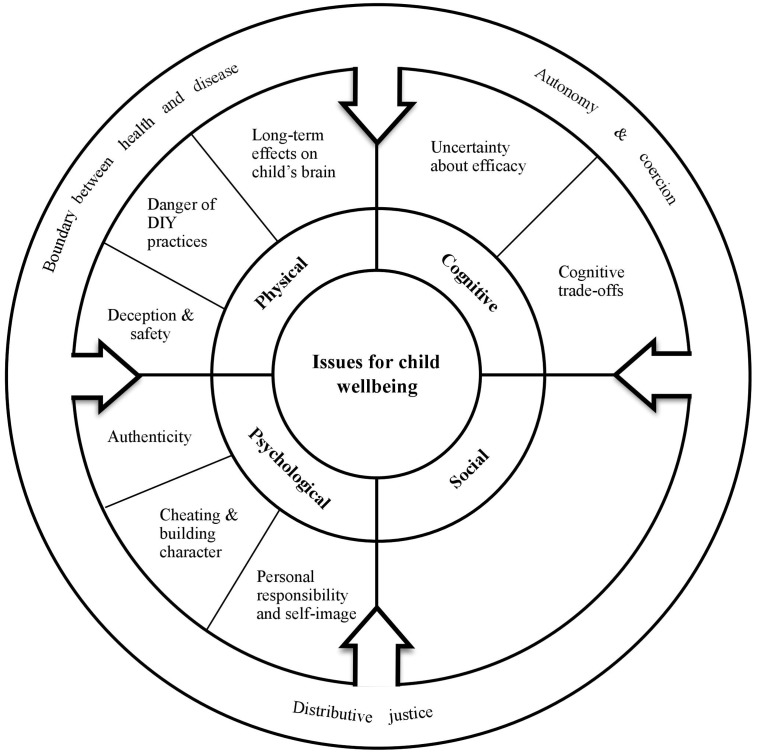
**Overview of the identified issues for child wellbeing that arise from the availability and education-related use of tES-based enhancers**.

Our review showed that the availability and education-related use of tES-based enhancers is associated with a wide range of issues that could directly or indirectly influence child wellbeing. **Figure 2** depicts the issues for child wellbeing that we identified in the literature, based on the main structure of our child wellbeing framework (see **Figure 1**). Although in this section, we link most issues to a particular child wellbeing domain, one must remember that child wellbeing is a holistic concept and that some issues might therefore actually span multiple domains or have close connections with issues in other child wellbeing domains. In addition, we would like to note that we did not identify any issues that directly relate to our definition of social wellbeing of the child, which is targeted at the individual level (see **Figure 1**). One possible explanation relates to our focus on enhancement practices that aim to improve *cognitive* functioning and school performances, instead of enhancement methods that are directly targeted at improving an individual’s social skills and social functioning. Evidently, we do not claim that tES is unable to directly affect social skills or an individual’s ability to function in social structures, but this particular enhancement effect was not frequently discussed in the selected literature (see Supplementary Materials). We did identify several issues that relate to the *societal context* in which children reside (i.e., depicted as the circle in **Figure 1**), and although these issues are not directly affecting the social functioning of an individual child, they do apply to social structures and dynamics (e.g., relationships, social ordering, interaction patterns) in the environment of the child that could indirectly impact each of the child wellbeing domains. The subsequent paragraphs will describe each of the issues that we identified in the literature.

### Issues for Physical Child Wellbeing

#### Safety and Long-Term Effects on the Child’s Brain

Safety of enhancement methods is a frequently addressed concern in the literature, and is an issue that could pose a serious threat to the child’s physical wellbeing. Authors expressed an optimistic view regarding the acute safety profile of tES techniques with acute side-effects that seemed rather mild (e.g., headaches, skin irritations). Yet, most of them also emphasized that caution should be warranted with regard to the long-term effects of tES-based brain stimulation, as little is yet known about such potential effects (Chatterjee, 2013; Dresler et al., 2013; Hildt, 2014; Lapenta et al., 2014; Maslen et al., 2014b).

In addition, special reference should be made to the current lack of translational studies in healthy children. Authors stress that established knowledge on potential side-effects and efficacy is derived from studies that have used adult participants, but that the developing brains of children might react differently to either pharmaceutical enhancing substances or tES technologies (Singh and Kelleher, 2010; Cohen Kadosh et al., 2012; Flanigan, 2013; Graf et al., 2013; Levy and Savulescu, 2014; Maslen et al., 2014b). Cohen Kadosh et al. (2012) specifically refer to tES-based neurotechnologies and explain that the localization of the correct stimulation sites might be problematic, since the child’s brain continuously develops and the location of stimulation sites might therefore change. Since guidelines for the use of tES technolgies in children are still lacking (Levy and Savulescu, 2014), the use of tES on children might result in unexpected side effects that could be detrimental for the child’s health. Flanigan (2013) further stresses that evidence on safety and side-effects of neuroenhancement in children will probably not become available any time soon, as the enrollment of healthy children in clinical trials is generally considered to be unethical. Although we could imagine that children with diagnosed cognitive impairments could be allowed to participate in future clinical trials, this still would not bring any certainty for the safety of tES-based technologies in *healthy* children. An atypical brain might react differently to stimulation than a typical brain (Cohen Kadosh et al., 2012), and the usage-frequency might be different for enhancement compared to treatment, which could result in disparate effects on the brain (Singh and Kelleher, 2010). Therefore, the current lack of translational studies on tES-based enhancement in healthy children augments the concerns about usage-safety.

#### The Danger of Do-It-Yourself (DIY) Practices

Another issue for physical safety of the child, particularly relevant in the case of tES, relates to the risks associated with do-it-yourself (DIY) practices of enhancement, either in the form of home-building practices of tES devices or the home-use of premanufactured tES-technologies by lay people. Fitz and Reiner (2014) explain that an “*easily accessable world of DIY tDCS enhancement*” has arisen due to the inexpensiveness of the technique and the easily accessible ingredients that are needed to build a tDCS device at home (p. 74). Consequently, lay people are enabled to use these stimulation devices without sufficient background knowledge on brain functionality or safe usage guidelines (Levy and Savulescu, 2014). Fitz and Reiner (2014) reason that the self-created tDCS devices allow individuals to manipulate a broad set of parameters, including polarity, current density, stimulation duration, and frequency of use. Moreover both Cohen Kadosh et al. (2012) and Fitz and Reiner (2014) express their concerns about electrode placement of premanufactured or home-built tDCS-devices, and the possible use of tDCS on cortical areas for which tDCS was not investigated. Since the safety of tES-based technologies has only been tested in clinical settings and effects of home-use, with potentially more extreme parameter settings, are still unknown (Fitz and Reiner, 2014; Hildt, 2014), the use of DIY tDCS by parents or minors at home or in school might put the child’s physical well-being at risk.

#### Deception and Safety

The use of deceptive terms and messages about cognitive enhancers was also considered an issue by some authors, as it might result in parents and children underestimating the possible safety risks of certain enhancement techniques. Concerns were expressed about both concealed and direct-to-consumer advertisement, and the unrealistic expectations of enhancement efficacy they may raise in parents and teachers (Singh and Kelleher, 2010; Flanigan, 2013). In addition, Cohen Kadosh et al. (2012) mention that people have the tendency to consider the use of external enhancers, which do not physically enter the body, to be less questionable than the use of internal enhancers that are taken up by the body, such as pharmaceuticals. As a consequence, people might unjustly assume that the use of tDCS is quite safe, considering its external mode of application (Cohen Kadosh et al., 2012). Lastly, both Fitz and Reiner (2014) and Hildt (2014) refer to the deceptive nature of the term “non-invasive stimulation,” which is often used to describe the practice of tDCS and other tES-technologies. This term is derived from the surgical literature, in which it is often used to differentiate tES-techniques from practices such as deep-brain-stimulation that require surgery (Fitz and Reiner, 2014). However, the term “non-invasive” might evoke a false sense of security, which could result in the underestimation of the safety risks involved in tES-based enhancement, and could therefore pose a threat to the wellbeing of children.

### Issues for Cognitive Child Wellbeing

#### Efficacy

Although tES is associated with a range of promising cognitive benefits, which could potentially boost children’s educational performances, many authors also refer to the current level of uncertainty when it comes to these cognitive results. Dresler et al. (2013), for example, explain that the efficacy of tDCS is highly dependent on the identification of the correct stimulation site, and that the most effective tDCS-enhancement studies have employed functional magnetic resonance imaging (fMRI) to guide the localization of stimulation sites. The fMRI-tool will not likely be part of the tDCS-set that individuals can use at home or in schools, and the enhancement effects of tDCS devices employed by inexperienced home-users might therefore be less pronounced. Similarly, reversing the polarity of a tDCS device could generate opposite effects on cognitive performance and thus decrease the intended effects of stimulation, as can be concluded from studies that Fitz and Reiner (2014) mention in their ethical analysis. Furthermore, Levy and Savulescu (2014) refer to studies that show the individual variability of tES-efficacy, with enhancement effects being more pronounced in subjects with low base-line performance compared to individuals with high base-line performance. However, it should be noted that alternative explanations might be possible for these individual differences, such as ceiling effects or regression to the mean. Overall, it seems that no certainty exists yet with regard to the benefits of tES-based enhancement for cognitive wellbeing, and incorrect application settings could even result in impairment of cognitive function. Cohen Kadosh et al. (2012) stress that this empirical uncertainty about potential positive or negative effects on cognitive functioning is even greater when it comes to tES-based enhancement of healthy children, as no scientific studies have yet been performed on this specific sub-population.

#### Cognitive Trade-offs

An issue that seems even more problematic for the cognitive wellbeing of the child than questions regarding the efficacy of tES-based enhancers is the concern for cognitive trade-offs. A cognitive trade-off refers to a functional increase within one cognitive domain that goes at the expense of a functional decrease within another cognitive domain. A hypothetical example would be an increase in numerical proficiency that goes at the expense of verbal word recognition. In their ethical article on non-invasive brain stimulation, Hamilton et al. (2011) suggest that the mechanisms of tDCS might be more prone to induce cognitive trade-offs than the intake of enhancing pharmaceuticals. They explain that the stimulated brain region could be responsible for more cognitive functions than the targeted one, which increases the chance of additional and unforeseen effects. Alternatively, they suggest that the stimulation of one brain area might result in the subsequent inhibition or excitation of other brain regions to which the stimulated area is functionally connected. The cognitive functions that are regulated by these indirectly manipulated brain regions could therefore become affected as well.

Although most authors of ethical literature who report on the issue of cognitive trade-offs explicitly refer to one or two clinical studies in which a trade-off effect was found (Hamilton et al., 2011; Cohen Kadosh et al., 2012; Hildt, 2014; Levy and Savulescu, 2014; Maslen et al., 2014b), Fitz and Reiner (2014) claim that these recorded trade-offs could just be the tip of the iceberg. They explain that most study protocols solely include measures for cognitive functions that were the main target of their enhancement procedures, and that unexpected effects on other cognitive domains could therefore have remained unnoticed. In order to see whether this could be the case, they performed a meta-analysis on 112 tDCS studies, of which the results were reported in their book chapter on ethical aspects of tDCS-based enhancement. When they grouped all cognitive effects that were found across studies with a similar mode of tDCS application, e.g., with the cathode (i.e., positive electrode) placed over the dorsolateral prefrontal cortex, their results showed that the same stimulation paradigm could indeed result in different effects. Stimulation with the cathode over the left dorsolateral prefrontal cortex, for example, caused impairment in declarative memory in one study, while it resulted in improvement of pleasant image recall in another. This supports the idea that tDCS-based enhancement might result in more cognitive trade-offs than we are currently aware of.

Some authors specifically refer to the issue of cognitive trade-offs with regard to tDCS application to children (Cohen Kadosh et al., 2012; Levy and Savulescu, 2014; Maslen et al., 2014b). They stress that stimulation of certain brain areas could disturb the typical development of a child’s brain, which might result in permanent trade-offs. If cognitive trade-offs appear to be common to neuroenhancement, doom scenarios as described by Maslen et al. (2014b), in which parents and children choose for enhancement, but inherently choose for impairment as well, might become reality. This could negatively impact the present-day cognitive wellbeing of children, but also their future cognitive wellbeing if the effects prove to be permanent.

### Issues for Psychological Child Wellbeing

#### Authenticity

A common issue discussed by ethicists in the field of neuroenhancement relates to the impact that cognitive enhancers, including tES technologies, have on human authenticity. This concept is described as *“an ethical ideal that allows the true development of unique individuality and self-fulfillment throughout life”* (Graf et al., 2013, p. 1257), which seems highly applicable to the psychological wellbeing of the child. Opinions with regard to this issue seem to divert, but although both pro- and counter arguments are put forward in the literature, not many authors actually take a specific stance.

Concerns about the potential harm that both pharmaceutical and tES-based enhancers could do to the authenticity of an individual user were related to the possible destruction of personality aspects that constitute to the true identity of a person. People tend to see only some human characteristics as relevant contributors to one’s personality and true identity. For instance, many might not consider a pure enhancement of memory-related skills or concentration as a change in personality, but do consider their indirect effects on virtues, such as honesty and fairness, as problematic for the preservation of one’s personal identity (Hamilton et al., 2011). Therefore, it is difficult to set clear boundaries as to what aspects of human being are morally acceptable to enhance, and what aspects are not (Hamilton et al., 2011). It could indeed be regarded problematic if the use of cognitive enhancers jeopardizes some of the most fundamental characteristics that distinguish us as human beings (Chatterjee, 2013), or if cognitive enhancement practices result in societal disregard for human gifts, talents, and achievements, and thus create a disregard for the true self (Hamilton et al., 2011). Issues of harmed authenticity carry even more weight when we consider the use of cognitive enhancers by *children.* Children are still developing and exploring their identity and they should have the opportunity to discover their own authentic self (Singh and Kelleher, 2010; Gaucher et al., 2013; Graf et al., 2013; Nagel and Graf, 2013). By allowing children to use cognitive enhancers, be it pharmaceuticals or tES-based devices, they could be deprived of the chance to create their own true identity and to develop their natural gifts and talents for their future lives (Singh and Kelleher, 2010; Gaucher et al., 2013). Therefore, not only the preservation of present authenticity was identified as a concern, but also the protection of future authenticity of the child (Nagel and Graf, 2013).

Besides the elaboration on the potential demolition of a child’s authentic self, arguments were identified that could mitigate the authenticity concerns. Some authors, for example, nuanced that for some individuals cognitive enhancement could be a tool to create a more authentic, instead of less authentic, version of the self (Flanigan, 2013; Graf et al., 2013; Nagel and Graf, 2013). Singh and Kelleher (2010), for instance, refer to a study, which shows that the use of stimulant medication by children diagnosed with ADHD has a positive influence on their perceived authenticity and experienced levels of agency, and Chatterjee (2013) uses the case of sex change operations to exemplify that changing an essential human characteristic could also promote the expression of the true and authentic self. Furthermore, some scholars stressed that the use of tES-technology will not lead to sudden acquisition of skills that one did not have before, which could unsettle your personal sense of identity (Cohen Kadosh et al., 2012; Levy and Savulescu, 2014). Instead they emphasize that the learning process still requires effort (Cohen Kadosh et al., 2012; Levy and Savulescu, 2014), and is thus comparable to other forms of educational guidance and teaching that are not considered harmful to a person’s authenticity (Levy and Savulescu, 2014). Their argument thus seems to appeal to a different enhancement scenario, in which tES-based technologies cannot create new talents or abilities, but can solely improve the natural capabilities of a person, which are already part of their authentic self.

Not all authors that tried to temper authenticity concerns did so by counterclaiming the potential harmful effects of cognitive enhancers for authenticity. Flanigan (2013) suggests that some people might not regard authenticity as important value, and thus do not concern themselves about this issue. She elaborates that even if someone would hold authenticity as a personal value, this value could still be overruled by other values, such as the desire to fit in. Her argument therefore seems to claim that the choice to protect one’s own authenticity should be a personal one, not a collective one.

#### Cheating and Building Character

A concern that is closely related to the authenticity issue is cheating. It revolves around the question whether one’s capabilities are still true to one’s authentic self after the use of a cognitive enhancer, and if its fair to work and study with enhanced capabilities. According to Chatterjee (2013), the extent to which the use of cognitive enhancers could be considered as a form of cheating depends on the relative weight that is put to end versus means of a certain task. If the outcome is considered more important than the preceding actions to achieve this outcome, then the use of an enhancer might not be regarded as a problem. However, if the learning processes were more important, taking the enhancer shortcut would diminish the worth of the eventual outcome, which would be defined as cheating and a mere reduction of ones efforts.

Sometimes the concerns about cheating and reduced efforts are also linked to worries about building character. Dresler et al. (2013) argue that they do not necessarily oppose to short cuts, but that the more effortful and unenhanced path can sometimes provide supplementary advantages, such as increased discipline, dedication, self-knowledge, and feelings of self-mastery. The avoidance of hardship and difficulties along the way can thus refrain people from obtaining such virtues and thereby weaken one’s character (Chatterjee, 2013). Gaucher et al. (2013) emphasizes that life lessons about the process of learning and its difficulties are especially important for children, who need to develop into resilient adults.

The concerns about cheating and depletion of character in the enhancement debate might be downplayed by the argument that current neuroenhancers still require effort in order to obtain successful results (Cohen Kadosh et al., 2012; Levy and Savulescu, 2014). Cohen Kadosh et al. (2012), for example, stress that up to now, tDCS has shown to be most effective when used in training paradigms, and similarly Levy and Savulescu (2014) emphasize that traditional learning processes remain important for profitable tES-induced enhancements. Thus, the use of tES-based technologies might not necessarily imply that effort is futile and hardship is avoided, i.e., easy short cuts are unlikely.

#### Personal Responsibility and Self-image

The use of cognitive enhancers by children and students also evokes questions regarding the effects on feelings of personal responsibility and the child’s self-image (Singh and Kelleher, 2010; Graf et al., 2013). Singh and Kelleher (2010) refer to studies, which show that children and their parents might attribute their achievements to the effects of the enhancer, instead of their own efforts, and that children could justify failures by explaining that they did not use their enhancing medications. Such a loss of personal responsibility could lead to psychological dependence on cognitive enhancers. In addition, some authors warn for the risk that children who use enhancing substances might consider themselves abnormal. This could result in a distorted self-image, especially if the child is also treated differently by people in his or her environment (Graf et al., 2013). Evidently, such feelings would be destructive to the psychological wellbeing of the child. On the other hand, the use of cognitive enhancers could also boost a child’s self-image. One can easily imagine how cognitive enhancers could increase self-confidence of children who experience cognitive struggles. Even if the cognitive effects would not be observed in more objective experiments, their placebo effect could still mitigate study-related worries (Flanigan, 2013). In this light, cognitive enhancers thus seem able to improve psychological wellbeing.

### Issues Related to the Societal Context

#### Autonomy and Coercion

The tension between autonomy and coercion is one of the issues associated with the societal context of the child. On the one hand, there is the autonomy of parent and child, including their right to freely decide whether they would like to use enhancers. On the other hand, there is the fear that widespread availability and use of enhancers creates coercive forces, which could actually confine their autonomy. With regard to the former, people often refer to the autonomy principle, which is highly valued in medical practice (Graf et al., 2013). This principle discloses the right of an individual to decide freely whether he/she would like to make use of a certain (risky) treatment or not, and is often used in the neuroenhancement debate to advocate the permission of cognitive enhancers for healthy individuals. However, when the issue regarding the free choice to enhance is shifted toward the context of a child, a difficult question emanates: to what extend should children be considered autonomous and thus capable of making their own decisions? (Singh and Kelleher, 2010; Chatterjee, 2013; Flanigan, 2013; Gaucher et al., 2013; Graf et al., 2013; Nagel and Graf, 2013; Maslen et al., 2014b).

Although most authors acknowledge that young children do not possess sufficient decision-making capacity to make enhancement-related choices, disagreement arises when specifically focusing on teenagers and adolescents, who are relatively close to maturity. Some authors refer to studies, which have shown that adolescents do not yet possess mature decision-making capabilities (Gaucher et al., 2013; Graf et al., 2013; Nagel and Graf, 2013), and are prone to impulsive actions or have a lack of insight regarding long-term implications (Gaucher et al., 2013). In contrast, other authors mention studies that have demonstrated adolescents’ capability to understand their medical condition (Singh and Kelleher, 2010; Chatterjee, 2013) and can make justifiable medical decisions (Chatterjee, 2013; Flanigan, 2013; Maslen et al., 2014b), which would support the idea that adolescents should be able to make autonomous decisions. Yet, the majority of these authors remain cautious, since cognitive enhancement might induce long-term effects, which may be difficult for adolescents to fully appraise (Singh and Kelleher, 2010; Chatterjee, 2013; Maslen et al., 2014b). Unremarkably, this resulted in different opinions regarding the extent to which the autonomy of the adolescent should be respected by parents, physicians, and societies. Gaucher et al. (2013) argue that the decision to use cognitive enhancers should not be made by the child, but by society that applies the best interest of the child principle. Singh and Kelleher (2010) do not agree that society should be deciding, but advocate an approach in which parents’ consent and children’s assent is both required to allow the use of cognitive enhancers. Flanigan (2013) and Maslen et al. (2014b) seem more lenient by suggesting that adolescents from the age of 16 should be considered autonomous (Maslen et al., 2014b), or even by stating that the autonomy of all teenagers should be respected, provided that physicians regard the child as autonomously capable (Flanigan, 2013).

Besides the questions that the availability of cognitive enhancers elicit regarding the child’s present state of autonomy, the use of enhancing substances and technologies also provokes questions regarding the future autonomy of the child and the right to an open future (Gaucher et al., 2013; Graf et al., 2013; Nagel and Graf, 2013; Maslen et al., 2014b). This right implies that children should have “*as many open options, opportunities, and advantages as possible*” upon entering their adult lives (Graf et al., 2013, p. 1254). Improved cognitive performance, induced by neuroenhancing pharmaceuticals and technologies, might open new future doors for the child (Cohen Kadosh et al., 2012; Flanigan, 2013). However, as discussed earlier in the section on cognitive wellbeing, chances are that the application of tES could also generate (permanent) cognitive trade-offs that in some way may limit the child’s future options (Maslen et al., 2014b). This could result in feelings of comprised autonomy when the child reaches adulthood, especially if the decision to enhance was made by parents, instead of the child itself (Maslen et al., 2014b). Furthermore, Maslen et al. (2014b) argue that if a child suffers from a clear deficit, the use of tDCS might indeed serve the best interest of the child, despite the cognitive trade-offs that might occur. Yet, when clear evidence for cognitive deficits is lacking, the protection of future autonomy should outweigh the wish for beneficence (Maslen et al., 2014b).

Besides the issues that revolve around ‘the freedom to enhance,’ there are also concerns about people’s ‘freedom *not* to enhance.’ Fears are expressed that the availability of neuroenhancers would lead to coercive environments, in which it would be difficult for individuals to refuse the use of cognitive enhancers (Singh and Kelleher, 2010; Hamilton et al., 2011; Chatterjee, 2013; Dresler et al., 2013; Flanigan, 2013; Gaucher et al., 2013; Graf et al., 2013; Lapenta et al., 2014). One can distinguish two types of coercion: explicit and implicit (Hamilton et al., 2011; Chatterjee, 2013; Lapenta et al., 2014). Explicit coercion concerns situations in which an individual is explicitly requested by their superiors or caregivers to use a cognitive enhancer for performance improvement (Chatterjee, 2013). Implicit coercion is more subtle and refers to a situation in which an individual feels pressured to use cognitive enhancers in order to satisfy the requirements of a high standard and often competitive environment (Hamilton et al., 2011). For instance, expanded availability of cognitive enhancers could lower acceptance levels of discernable deviations in cognitive functioning and thereby implicitly give children the impression that they should push themselves to exceed their natural potentials (Singh and Kelleher, 2010). This in turn might indirectly affect their psychological, physical or cognitive wellbeing. The issue of coercion seems particularly relevant with regard to children, as they are not yet accredited full legal autonomy, and could therefore be even more vulnerable to external forces in ambitious climates (Chatterjee, 2013; Graf et al., 2013).

In the literature, three main sources were identified that were particularly relevant for possible coercion of children: parents, educational environments, and peers. The first one, parents, is highly applicable to school-aged children who still live with their families. Several authors expressed their concerns about parents who might explicitly encourage their children to use cognitive enhancing substances, be it with the best intentions for their child (Flanigan, 2013) or out of the desire to develop exceptionally successful children at all costs (Singh and Kelleher, 2010; Chatterjee, 2013). In some cases, parental wishes to enhance their children might be pushed by Western conceptions of good parenting, which require parents to take every measure possible to secure the prosperity of their child (Singh and Kelleher, 2010). Besides success-driven motivations for parental pressure, Singh and Kelleher (2010) also highlight the possibility that disorganized and less-resourceful households might use cognitive enhancers as a “*child management tool,”* which allows parents to refrain from improving parenting practices and home conditions (p. 9). The consequent coercive forces from parents toward their children could translate themselves into psychological and emotional distress of the child (Singh and Kelleher, 2010).

The second source of pressure that was identified in the literature was the educational environment, including schools. Graf et al. (2013) mention that teachers often praise enhancing stimulants for their valuable effects, and Chatterjee (2013) suggests that teachers are not hesitant to talk parents into purchasing pharmaceuticals to improve their child’s performance in school. Some schools would even denounce parents to disregard their child’s educational development when ignoring the advice to use pharmaceutical enhancers (Singh and Kelleher, 2010). Teachers’ motivations to openly and explicitly promote the use of cognitive enhancing substances could be related to a desire to help individual children succeed, or teachers might want to find an easy way to control a class room full of kids (Singh and Kelleher, 2010). This type of teacher-induced pressure seems to work mainly via parents.

The final source of pressure is the use of cognitive enhancers by peers, which could implicitly encourage individuals to take part in similar behaviors. School-aged children, for instance, might be susceptible to trends in their environment, including the use of cognitive enhancers by their peers, and might therefore decide to start using these substances as well (Graf et al., 2013). Explicit and implicit social factors could thus hinder children’s ability to maintain their autonomous stance in their decision to enhance, and might therefore pose a threat to the child’s present and future psychosocial wellbeing.

#### Distributive Justice

Another question that is often raised in the neuroenhancement debate is: who will have access to new cognitive enhancement techniques, and who can experience the cognitive benefits? That is, *if* these benefits can truly be achieved. It is a question related to the value of distributive justice, and opinions on this matter differ. Many envision that the availability of cognitive enhancers, be it pharmaceuticals or stimulation devices, would evoke inequalities and widen the gap between the rich and the poor (Lev, 2010; Hamilton et al., 2011; Dresler et al., 2013; Gaucher et al., 2013; Graf et al., 2013). Since cognitive enhancers differ from commonly acknowledged treatments, health insurance companies will probably refrain from covering their costs (Hamilton et al., 2011; Chatterjee, 2013; Lapenta et al., 2014). Therefore, only those people that can afford neuroenhancers will have access to them and the opportunity to profit from their potential cognitive benefits. In addition, Lev (2010) suggested that affluent citizens would be more likely to know of existing neuroenhancers and would have more time to obtain them.

Although many authors recognized increased societal disparities as problematic, some of them also argue that this would probably not be a decisive issue in the neuroenhancement debate, as throughout the history access-related inequalities have widely been accepted on all sorts of levels, including nutrition, shelter, education and medical care (Hamilton et al., 2011; Chatterjee, 2013). In addition, Dresler et al. (2013) plead that natural enhancers, such as sleep and exercise, are just as likely to generate equality issues. As an example they use a physically disabled individual that is unable to benefit from the enhancing merits of exercise. Thus, according to these authors, the availability of neuroenhancers does not necessarily produce new justice-related issues, but merely fits into a society that is already filled with social inequalities.

In contrast to the pessimistic voices that warn for increased disparities, several authors predict an opposite effect on distributive justice and explain that the availability of cognitive enhancers could result in more equality. However, these arguments seem related to equality of opportunity, and presume a situation in which equal access to enhancers is already accomplished. Flanigan (2013), for example claims that neuroenhancing substances could compensate for already existing inequalities, such as differences in school quality, and provide children the opportunity to succeed in difficult environments. In such a scenario, the author thus assumes that children in difficult environments have full access to enhancing technologies. Similarly, Levy and Savulescu (2014) point toward evidence, which shows that both pharmaceutical enhancers and tDCS are most effective in people with a low performance base-line, and claim that this could result in increased equality of opportunity. The issue of access, which actually precedes the issues related to equality of opportunity in later life, is not discussed. Nevertheless, Fitz and Reiner (2014) and Lapenta et al. (2014) do seem to have targeted their positive expectations toward equality of access, although mainly with regard to the availability of tDCS. They claim that tDCS is a relatively cheap technique compared to the long-term use of pharmaceuticals or other complex technologies, and is consequently also accessible for less resourceful people. Yet, it seems that without money, one is still unable to buy a tDCS device. Therefore, it seems more appropriate to state that tES-devices are relatively more accessible compared to pharmaceuticals, although they still require people to have money. For those who cannot afford the use of tES-based enhancers, absolute cognitive performance will not be negatively affected. However, the relative cognitive performance decreases when other children, who do have the resources to apply tES-based techniques, enhance themselves. This could result in a diminished self-concept of cognitive ability or relatively lower educational achievements, which both constitute to the cognitive wellbeing of the child.

#### Boundary between Health and Disease

The distinction between health and disease or enhancement and treatment was a reoccurring theme in a diverse set of articles, and evoked questions with regard to the use and availability of cognitive enhancers. Two main concerns could be distinguished. First is whether we should use the distinction between health and disease to judge if a child should be allowed to use enhancers (Flanigan, 2013; Gaucher et al., 2013; Levy and Savulescu, 2014; Maslen et al., 2014b). Since tES devices are currently still unregulated and do not require a doctor’s judgment and prescription, this question is an important one in considering potential governance measures. The second concern was related to a potential shift in the social perception of what constitutes health and disease. Authors referred to the risk of medicalization of normal traits (Graf et al., 2013; Levy and Savulescu, 2014), and although this specific term is obviously linked to the medical system, it can easily be translated to the case of unregulated tES-based enhancement devices. Children and their parents might not search for an official diagnosis to gain access to enhancers, but the widespread availability and use of tES-devices could still shift the general image of what is considered to be a “normal” performance level. One can imagine that a shift in the boundary between “normal” and “abnormal” might result in parents and children accepting higher risks to reach a certain performance level, and thereby disregarding potential threats for child wellbeing.

## Understanding the Complexity of the Issues for Child Wellbeing

Our literature review shows that the availability and use of tES-based enhancers might result in a high number of issues for child wellbeing that together form a complex web of uncertainties and concerns (**Figure 2**). However, in order to explore our subsequent question on “how to deal with the complexity of issues in a responsible way?” We first need to elaborate on the character of the issues that we described, and explain what makes them so complex.

When looking at the issues we have described in the previous section, we recognize two main factors that contribute to the complex character of the issues: (1) high levels of factual uncertainty, and (2) high levels of moral diversity. With regard to the factor of uncertainty, we observe that many empirical gaps are still present in the neuroscientific field; no clarity has been established yet on the long-term (side) effects of tES-based enhancement or on the technique’s influence on the developing brain. Moreover, little is known about the exact magnitude and interpersonal variability of the enhancement effects, and questions about potential cognitive trade-offs and psychological and societal effects have still largely remained unanswered. As a consequence, it is difficult to determine the actual benefits and risks of the enhancement technique.

The second factor that adds to the complexity of the issues surrounding tES is the presence of moral diversity. Different moral views exist on whether a certain enhancement effect is desirable and whether potential risks are acceptable. These views depend on one’s personal perspective, i.e., the underlying structure of beliefs, values and assumptions that frames one’s opinion (Schön and Rein, 1994; Kupper and De Cock Buning, 2011). When considering the described issue of cheating, for example, we could argue that the relative weight that one puts to the end versus the means of a certain task, influences whether we believe that it is acceptable to use tES for accomplishing that particular task. Similarly, we could ask ourselves: do I consider the child’s authenticity to be more important than the child’s ability to fit into the group, or than the opportunity to freely decide to use cognitive enhancers? What is the relative importance that I ascribe to the fair distribution of goods and services in society? And do I think that certain risks are just as acceptable for healthy children as they are for children with a disorder? One’s answer and stance will depend on the values that one holds dear, and the weight that one attributes to them, which is likely to be different for each individual.

Besides diversity in moral views due to different weighing of values, people could also interpret values differently. When considering the issue of autonomy and coercion, we see that autonomy could be interpreted as the right to freely decide to make use of enhancers, or as the right to freely decide *not* to make use of enhancers. If one focuses on the first interpretation of autonomy, then one might not consider the wide availability and use of tES-devices as problematic. However, when one focuses on the second interpretation, then the wide availability and use of tES might all of a sudden be considered a threat, as it could create coercive environments in which children might be pressured to apply these techniques against their own will. Likewise, we encountered different value interpretations with respect to the authenticity issue that we described. Some seem to interpret authenticity as the degree to which one remains true to his or her “natural” characteristics, and therefore condemn the use of enhancing techniques, while others seem to suggest that authenticity can actually benefit from a little outside help, as this can promote authenticity to come to full expression.

Interaction between factual uncertainties and moral diversity further complicates the issues surrounding tES. Uncertainties about effects and impact, for instance, can lead to speculation in the neuroenhancement debate. Chances exist that different people use arguments that apply to different speculations about consequences, and thus different enhancement scenarios (e.g., “enhancement as the sudden acquisition of skills that one did not have before” versus “enhancement as a process that still requires effort and only improves one’s natural capabilities”). This may influence the stances toward tES-based enhancement and could make the various arguments difficult to compare.

It is clear that scientific uncertainties and all the different interpretations and stances obscure the development of clear and straightforward policy answers. Nevertheless, tES-devices will further penetrate the market and will become increasingly available to consumers, including school children and their parents (Maslen et al., 2014a). Therefore, the challenge for policy-makers resides in how they will deal with the high levels of uncertainty and moral diversity that surround the emergence of tES-based technologies for enhancement in children.

## Toward an Approach of Responsible Innovation Governance

In the past, the lack of scientific knowledge regarding the effects of emerging technologies has often been used as an excuse for regulatory passivity (Martuzzi and Tickner, 2004). Only when abundant empirical evidence would indicate that a specific technology or product could be harmful, policies would be created to prevent further negative consequences (Martuzzi and Tickner, 2004). However, the rapid growth of complex technological innovations has bolstered the realization that science is not always able to discern a technology’s full range of (societal) consequences in advance, and that a “wait and see” policy approach could lead to societal damage and high public costs. Many therefore stress the importance of a precautionary attitude toward technologies with uncertain impacts, and call for early risk intervention in order to avert irreversible negative consequences (Renn and Klinke, 2015)^2^. This review has shown that the availability and education-related use of tES-based technologies by healthy high school children are associated with various potential – but uncertain – risks. In our view, the fact that these risks involve the wellbeing of children should unquestionably be an extra incentive to apply a certain level of precaution and anticipatory behavior. Yet, mere focus on precaution disregards that uncertainty is partly unavoidable when dealing with new and emerging technologies, and can even refrain us from discovering more about the actual risks and benefits that are generated by the use of tES. For this reason, some researchers suggest to treat new and emerging technologies as ongoing social experiments, whose acceptability requires sustained scrutiny (Taebi et al., 2012; van de Poel, 2013).

When focusing on the case of tES-based enhancement in children, it is evident that scientific uncertainty is not the only factor that needs to be dealt with. The high level of moral diversity is a factor just as compelling, and might even be more crucial for the identification of appropriate policy responses to this newly emerging technology. This is illustrated by work of Renn et al. (2011), who promote the use of a precaution-based governance style when confronted with scientific uncertainty about impacts, but also state that when moral diversity – or “ambiguity,” as they phrase it – is encountered, the participative character of the governance approach becomes most imperative. They therefore recommend a discourse-based governance style, based on meaningful dialogue between all stakeholders and affected publics, when dealing with issues of both scientific uncertainty and moral diversity. Their suggestion corresponds to the broader move toward participation that we have encountered since the 1960s in the field of science and technology governance, and which, since then, has been embraced by various scientific traditions, including Technology Assessment (Schot and Rip, 1996; Palm and Hansson, 2006), Public Engagement (Rowe and Frewer, 2005; Delgado et al., 2010), Anticipatory Governance (Guston, 2014), Risk Governance (Klinke and Renn, 2002; Renn et al., 2011), and Responsible Research and Innovation (Owen et al., 2012). These traditions emphasize that participatory practices stimulate mutual respect between people with different perspectives, and contribute to the creation of a collective understanding about the values at stake. And although this does not necessarily assure the provision of straightforward answers, it does have most potential to contribute to the construction of governance measures that are more broadly accepted in society (Renn et al., 2011).

In line with both the call for ongoing scrutiny of new technologies used in society, and the trend of increased public and stakeholder participation in the field of science and technology, we argue for an approach of responsible innovation governance to deal effectively with the issues concerning tES-based enhancement by healthy minors. Now, an important question to touch upon is: what does such an approach entail? We will briefly elaborate on some aspects that – in our view – constitute to a framework of responsible research and innovation.

First is inclusive deliberation, which involves the assembling of and conversational exchange between various stakeholders and publics who do not usually interact, but nevertheless all have a stake in the issues at hand (de Jong et al., 2016). The education-related use of tES-based enhancers by minors is a multifaceted problem and its mitigation thus requires the integration of multiple knowledge traditions and perspectives. In other words, it is not a problem that responsible policy-makers or concerned neuroscientists alone can solve. The issues that we identified in this paper are, for example, intricately interwoven with society’s views on practices of education, child rearing and development, and the systems in which we envision our children to function as prospective adults. Solving the problem therefore begins with making an inventory of which perspectives and actors are to be involved. This paper already points to a few of them, being parents, schools, policy-makers, scientists, minors in school, national and international authorities, tES-producing industries, and the tES do-it-yourself community.

Second, deliberative practices should encourage both anticipation and reflection (i.e., sense-making). Based on current dynamics in research and innovation and our collective expectations, we can try to envision the future and the way tES devices might influence it. Evidently, predicting the future is a futile effort and most certainly not what anticipation is about (Guston, 2014). Instead, anticipatory activities aim to collectively *explore* a range of possible and plausible futures that could help us to increase resilience, and stimulate public dialogue on how to act in face of certain developments (Boenink et al., 2010; Stilgoe et al., 2013). In order to identify which actions should be taken to guide us to desirable worlds, we should also engage in reflective processes (Stilgoe et al., 2013; Kupper et al., 2015) that stimulate thought on current practices, the goals we strive for, the norms and values we hold dear, and the norms and values that institutional protocols and cultures communicate outward. For instance, one could imagine that schools consider both learning processes and outcomes important, but when only the learning outcomes are rewarded with grades, children and parents might get the idea that school only revolves around these, and start to adapt their behavior toward that end. By performing reflection, we become more aware of our current role and the ways we can influence future practices.

Scenario exercises prove to be a useful tool to stimulate anticipation and reflection in deliberative processes. Both Boenink et al. (2010) and Lucivero et al. (2011) have described methods to systematically develop plausible scenarios. These so-called “techno-ethical” scenarios provide detailed stories on how technology and society might co-develop over time, resulting in various types of futures. The scenarios help actors to imagine and discuss a broader range of impacts than they might initially think of, and at the same time prevent them from slipping into discussions that are merely based on science fiction (Boenink et al., 2010; Lucivero et al., 2011). We believe that the use of techno-ethical scenarios will also prove to be valuable in deliberations on tES-based enhancement. They will not only stimulate public discussion on the desirability of education-related enhancement practices of children using tES, but also provide sufficient contextual information to collaboratively identify when and where interventions and regulatory measures might be needed.

Thirdly, deliberation and anticipation by different parties is only meaningful when it can lead to action. This willingness, but also the ability to act differently is perhaps the most difficult, especially because of the presence of so many involved parties in the inclusive deliberation processes we are outlining here. As we are dealing with a new phenomenon (i.e., education-related use of tES-based enhancers), including new parties, it also means that there are no yet-defined role responsibilities to undertake certain actions. Therefore, we do not only collaboratively need to identify appropriate actions, but should also foster stakeholders’ willingness to act. This is an issue of paramount importance as with new innovations, identified actions usually run counter to existing institutional borders and forces (Kloet et al., 2012; Arentshorst et al., 2014). In the field of Responsible Research and Innovation recent work has started to identify how true responsiveness of actors can be promoted (de Jong, 2015). One way to stimulate action and responsive attitudes is to guide deliberative processes toward the formation of so-called communities of practice (Wenger, 2000). These are communities in which various actors (e.g., schools, policy-makers, DIY-community, scientists, parents, etcetera) with a shared domain of interest exchange information and experience through sustained interaction. It encourages mutual learning and the continuous production and implementation of new and creative solutions.

In the case of tES-based enhancement, a sustained interaction process should prevent a mere focus on conventional governmental solutions, such as restrictions on the sale of tES devices, the compulsory specification of potential risks and benefits on pre-manufactured devices (Maslen et al., 2014a), or the funding of extra research into the working-mechanisms and effects of tES-based enhancement. Although each of these measures may be valuable to employ, one should also explore the role responsibilities of other actors, and find ways to integrate “alternative” action strategies with governmental measures. In a deliberative process, stakeholders might, for example, collaboratively identify a role for the Do-It-Yourself community, in which representatives of this community are asked to help stimulate a shared responsible mindset amongst Do-It-Yourselfers (Fitz and Reiner, 2013). Schools may also find out that they could play a role, for instance by educating parents and teachers on the ethical dilemmas related to enhancement practices (Singh and Kelleher, 2010), or by reconsidering the way good educational performance should be assessed. With respect to the latter, one could image that a more balanced focus on both grades and virtues related the act of studying itself (i.e., the learning process) might mitigate the felt need to use enhancers. Another form of collaborative action – more targeted at the research level – is participatory agenda setting. Through a collective process of inquiry and deliberation, research priorities can be set that represent multiple interests and perspectives. These may serve as a “responsible” guiding structure for future explorations and studies on tES. Evidently, all the actions described here are merely examples, and we do not claim that these are the actions that *should* be undertaken. Eventually, it should be up to the collaborative efforts of all relevant stakeholder groups to explore, identify and weigh those solutions that they regard as most appropriate.

Lastly, we would like to stress that the deliberative search for solutions has a highly iterative character and requires continuous accommodation of progressive insights. New research findings might emerge that either decrease or increase the existing factual uncertainties surrounding tES, and thereby influence our stances toward the acceptability and desirability of the enhancement technique. Moreover, our moral standards are also subject to change (Swierstra et al., 2009), which implies that the value one places on, for instance, education is not necessarily stable over time. To accommodate these changing scientific uncertainties and morals, we need some degree of flexibility. For this reason, a one-off policy for tES enhancement seems inappropriate. Rather, an approach of *governance* is required that employs various instruments to target different groups at different levels (e.g., governmental, institutional, organizational, individual). This requires the involvement of various actors, each having their own instruments and sphere of influence. Not only is it crucial to allow these instruments to be iteratively adjusted over time, but they should also form a coherent whole to prevent fragmentation of the problem and to establish an effective overall approach.

The education-related use of tES-based enhancers by healthy minors is associated with a wide range of issues for child wellbeing, which are mainly incited by both factual uncertainty and moral diversity. In order to deal properly with the issues for child wellbeing, we recommend the use of a governance framework for responsible innovation. Through sustained interactions and co-production amongst various actors, this framework allows us to balances the different moral perspectives regarding tES use by minors, and to remain adaptive toward emerging knowledge and dynamic societal values.

## Author Contributions

The review analysis was conceived, performed and processed into a manuscript by JS under supervision of IdJ and NvA. Both IdJ and NvA contributed to the development of the research approach, and supervised the interpretation and structuring of data. Both IdJ and FK made major contributions to the discussion section in which the outcomes of the analysis are linked to the field of science and technology. IdJ, NvA, and FK all critically appraised the intellectual content and structure of the manuscript. All authors approved the final version of the manuscript, and agree to be accountable for all aspects of the work in ensuring that questions related to the accuracy or integrity of any part of the work are appropriately investigated and resolved.

## Conflict of Interest Statement

The authors declare that the research was conducted in the absence of any commercial or financial relationships that could be construed as a potential conflict of interest.

## Footnotes

1 Underwood, E. “Cadaver study casts doubt on how zapping brain may boost mood, relieve pain”, *Science*, April 20, 2016, accessed June 20, 2016, http://www.sciencemag.org/news/2016/04/cadaver-study-casts-doubts-how-zapping-brain-may-boost-mood-relieve-pain

2 European Commission (2000) *Communication from the Commission on the precautionary principle* (white paper 52000DC0001). Retrieved from: http://eur-lex.europa.eu/legal-content/EN/ALL/?uri=CELEX:52000DC0001
