# Supplementary material for: Transcranial Electrical Stimulation to Enhance Cognitive Performance of Healthy Minors: A Complex Governance Challenge
Source: Front Hum Neurosci. 2017 Mar 27;11:142. doi: 10.3389/fnhum.2017.00142 (PMC5366312; doi:10.3389/fnhum.2017.00142)
Supplement: Supplementary file 1 [file Table_1.docx]

Supplementary Material

**Transcranial Electrical Stimulation to enhance cognitive performance of healthy minors: A complex governance challenge**

Jantien Willemijn Schuijer, Irja Marije de Jong, Frank Kupper, Nienke Mariëlla van Atteveldt

*** Correspondence:** Jantien Willemijn Schuijer: j.w.schuijer@vu.nl

# Supplementary information on systematic search strategy

In January 2015 we performed a systematic search in three indexed databases: Pubmed, Web of Science and the Worldcat book database. Syntaxes for Pubmed and Web of Science consisted of two conceptual search elements: the word “neuroenhancement” or a related term, and a word such as “ethical”, “debate”, “issues”, “argument” or one of their synonyms (field: *Title* and *Abstract* in Pubmed, *Topic* in Web of Science). In Worldcat two simple search terms were applied to search within book titles: “neuroenhancement” and “cognitive enhancement”. More extensive search syntaxes did not yield higher numbers of relevant books. The search generated *n=1133* literature sources (508 Pubmed, 549 Web of Science, 76 Worldcat). A full overview of search syntaxes is presented in Supplementary Table 1.

We were interested in two types of relevant literature sources: 1) literature on the ethics of tES-based neurotechnologies, and 2) literature on the ethics of (mainly pharmaceutical) neuroenhancers specifically studied from a child’s perspective. After an initial screening of titles and a subsequent removal of duplicates 262 articles were selected for a review of abstract. A total of *n* = 244 sources were assessed as incompatible, and a consecutive full-text review resulted in the exclusion of an additional *n* = 6 sources. For the assessment of both abstracts and full-text bodies we used a set of inclusion and exclusion criteria (see Supplementary Table 2 and 3.). These criteria were also used in an additional hand-search, which resulted in the inclusion of 3 extra articles. The final literature selection consisted of 15 literature sources (9 related to ethics of tES, and 6 related to ethics of (pharmaceutical) enhancers studied from a child-perspective, see Supplementary Table 4.).

| **Supplementary Table 1. Search syntaxes per database** | | |
| --- | --- | --- |
| **Data base & search date** | **Search specifications** | **Search syntax** |
| PubMed  February 2, 2015 | Title and Abstract [Title/Abstract]  Time span: from 2005 to 2015 | [Neuroenhancement OR neuroenhancers OR neuroenhancing OR cognitive enhancement OR cognitive enhancers OR neuroenhancing technologies OR neurotechnologies OR neural enhancement OR performance enhancement OR brain augmentation OR cognitive augmentation OR neuro-enhancement OR neuro-enhancers OR neuro-enhancing OR enhance cognitive performance OR performance enhancing drug OR pharmacological enhancement OR perfomance enhancing technologies OR enhancement devices OR human enhancement]  AND  [Experience OR experiences OR opinions OR opinion OR reasons OR reason OR attitude OR attitudes OR perspectives OR perspective OR motive OR motives OR justification OR debate OR ethical OR ethics OR issues OR legal OR societal OR social OR neuroethics OR neuroethical OR neuroethically OR ethically OR argument OR arguments OR objection OR objections]^[[1]](#footnote-1)^ |
| Web of Science  February 4, 2015 | Topic [TS]  Time span: from 2005 to 2015 | [Neuroenhancement OR "cognitive enhancement" OR "human enhancement" OR "pharmacological enhancement" OR "enhancing technologies" OR "brain augmentation" OR "enhancement debate"]  AND  [Neuroethics OR neuroethical OR neuroethically OR argument OR arguments OR objection OR objections OR ethically OR experience OR experiences OR opinion OR opinions OR reasons OR reason OR attitudes OR attitude OR perspectives OR perspective OR motive OR motives OR justification OR debate OR ethical OR ethics OR issues OR legal OR societal OR social OR discussion]1 |
| WorldCat  February 10, 2015 | Title [Ti] and subject [Su]  Time span: 2005 -2015 | Neuroenhancement OR “cognitive enhancement” |

| **Supplementary Table 2. Inclusion and exclusion criteria for literature on ethics of tES-based neurotechnologies** |
| --- |
| **Inclusion criteria** |
| - Use by healthy individuals (children or adults) - Use for purpose of cognitive enhancement - tES-based technologies (tDCS, tRNS, tACS) - English language - Books and peer-reviewed articles published between 2005 and 2015 |
| **Exclusion criteria** |
| - Use by elderly or people with a disorder (psychiatric or somatic) - Deep brain stimulation, TMS, biotechnologies, eugenics, brain implants, or training - Use for purposes related to military environment, medical professionals, or sports - Experimental or clinical studies - Non-western perspectives (e.g. Japan, Korea, Buddhist) - Use for purpose of moral, or emotional enhancement - Book reviews |

| **Supplementary Table 3. Inclusion and exclusion criteria for literature on ethics of pharmaceutical enhancers, with child-centered perspective** |
| --- |
| **Inclusion criteria** |
| - Use by healthy children (< 18 years) - Use for purpose of cognitive enhancement - Pharmaceutical enhancers, or other type of neuroenhancer to improve cognitive abilities - English language - Books and peer-reviewed articles published between 2005 and 2015 |
| **Exclusion criteria** |
| - Use by university students or adults - Use of non-medical substances (caffeine, smoking, alcohol) - Deep brain stimulation, biotechnologies, eugenics, brain implants, or training - Experimental or clinical studies - Non-western perspectives (e.g. Japan, Korea, Buddhist) - Use for purpose of moral, or emotional enhancement - Book reviews |

| **Supplementary Table 4. Overview of selected literature sources** | | | |
| --- | --- | --- | --- |
| **Author(s)** | **Type of source** | **Main focus^[[2]](#footnote-2)^** | **Type of enhancers discussed** |
| Chatterjee (2013) | Book chapter | Ethics related to tES | TMS, tDCS, prescription drugs |
| Cohen Kadosh (2012) | Journal article | Ethics related to tES | tDCS |
| Dresler et al. (2013) | Journal article | Ethics related to tES | tDCS, TMS |
| Fitz & Reiner (2014) | Book chapter | Ethics related to tES | tDCS |
| Flanigan (2013) | Journal article | Ethics related to pharmaceutical enhancers, child perspective | Stimulant prescription drugs |
| Gaucher et al. (2013) | Journal article | Ethics related to pharmaceutical enhancers, child perspective | Stimulant and non-stimulant prescription drugs |
| Graf et al. (2013) | Journal article | Ethics related to pharmaceutical enhancers, child perspective | Stimulant prescription drugs |
| Hamilton et al. (2011) | Journal article | Ethics related to tES | tDCS, TMS |
| Hildt (2014) | Journal article | Ethics related to tES | tDCS |
| Lapenta et al. (2014) | Journal article | Ethics related to tES | tDCS |
| Lev (2010) | Journal article | Ethics related to enhancers, child perspective | Neuroenhancers (not specified) |
| Levy & Savulescu (2014) | Book chapter | Ethics related to tES | tES |
| Maslen, Earp et al. (2014) | Journal article | Ethics related to tES, child perspective | TMS, tDCS |
| Nagel & Graf (2013) | Journal article | Ethics related to pharmaceutical enhancers, child perspective | Stimulant prescription drugs |
| Sing & Kelleher (2010) | Journal article | Ethics related to pharmaceutical enhancers, child perspective | Stimulant prescription drugs |

1. Due to progressive insights and the specific purpose of this review in relation to the research topic, we eventually decided to remove papers from our literature selection that were related to motives, experience, attitudes and reasons regarding the use of cognitive enhancers. [↑](#footnote-ref-1)
2. The term “main focus” relates to the two types of literature sources that we considered relevant for this review: 1) sources that discuss the ethics of tES, and 2) sources that discuss ethics of neuroenhancers (i.e. pharmaceuticals in most cases) and that specifically apply a child perspective. [↑](#footnote-ref-2)
